# Predicting the victims of hate speech on microblogging platforms

**DOI:** 10.1016/j.heliyon.2024.e40611

**Published:** 2024-11-26

**Authors:** Sahrish Khan, Rabeeh Ayaz Abbasi, Muddassar Azam Sindhu, Sachi Arafat, Akmal Saeed Khattak, Ali Daud, Mubashar Mushtaq

**Affiliations:** a Department of Computer Science, Quaid-i-Azam University, Islamabad, Pakistan; b Department of Computer Science, University of Warwick, Coventry CV4 7AL, UK; c Faculty of Computing and Information Technology, King Abdulaziz University, Jeddah, Saudi Arabia; d Faculty of Resilience, Rabdan Academy, Abu Dhabi, United Arab Emirates; e Department of Computer Science, Forman Christian College (A Chartered University), Lahore, Pakistan

**Keywords:** Social media, Hate speech, Twitter, Machine learning, Prediction

## Abstract

Hate speech constitutes a major problem on microblogging platforms, with automatic detection being a growing research area. Most existing works focus on analyzing the content of social media posts. Our study shifts focus to predicting which users are likely to become targets of hate speech. This paper proposes a novel Hate-speech Target Prediction Framework (HTPK) and introduces a new Hate Speech Target Dataset (HSTD), which contains tweets labeled for targets and non-targets of hate speech. Using a combination of Term Frequency-Inverse Document Frequency (TFIDF), N-grams, and Part-of-Speech (PoS) tags, we tested various machine learning algorithms, Naïve Bayes (NB) classifier performs best with an accuracy of 93%, significantly outperforming other algorithms. This research identifies the optimal combination of features for predicting hate speech targets and compares various machine learning algorithms, providing a foundation for more proactive hate speech mitigation on social media platforms.

## 1 Introduction

While social media facilitates global communication and opinion exchange, it also poses challenges, including antisocial behaviors like trolling and hate speech [1], highlighting the need for effective mitigation strategies. Misusing the openness of social media platforms has become a major issue not only on specific microblogging platforms but also globally, necessitating action at both organizational and national levels [2]. Hate speech, in particular, can have severe consequences, potentially inciting real-world violence and further exacerbating social tensions [3].

Existing research predominantly focuses on the detection of hate speech content within social media posts. However, a critical aspect that remains underexplored is the prediction of which users are likely to become targets of hate speech. Identifying these potential targets can allow for proactive measures to warn and protect users before any harmful speech is directed at them.

Moreover, not only harmful content but also general facts, non-harmful content, or positive content can be targeted by hate users, especially if the person is famous. For example, even a post about a good deed, like a donation, can be targeted for hate. Other examples include tweets from politicians or actors who occasionally become part of a controversy, such as a politician's announcement of a policy change or an actor's post about a charitable event. These types of posts can attract hate despite their positive or neutral content. It is therefore important to identify not just the hate content but the potential targets of such content. In this study, we did not include data from verified (and potentially famous) users.

Predicting potential targets is important because it allows us to warn users about the risks associated with their posts before any hate speech is directed at them. This proactive approach differs from existing hate speech detection methods, which typically focus on identifying and removing harmful content after it has been posted. By predicting potential targets, we can mitigate the impact of hate speech and protect users more effectively. This approach helps in taking preventive measures rather than reactive measures, thus improving user safety and experience on social media platforms.

This research proposes a novel method to predict whether a particular post would be the target of hate or not. By predicting potential targets, users can be warned and better protected, which goes beyond merely identifying and removing hate content. We propose a novel Hate-speech Target Prediction Framework (HTPK) and introduce the Hate Speech Target Dataset (HSTD). The HSTD is the first dataset specifically designed to study posts that lead to users becoming targets of hate speech. Our framework utilizes a combination of TF-IDF, N-grams, and Part-of-Speech (PoS) tagging for analyzing and predicting hate speech targets with high accuracy.

The contributions of this research are as follows:

1. Development of the HSTD dataset, which contains tweets labeled to indicate whether they led to users becoming targets of hate speech.
2. Introduction of the HTPK framework, which predicts whether a user will become a target of hate speech based on their own tweets.
3. Investigation of the optimal combination of features for predicting hate speech targets.
4. Comparative analysis of various machine learning algorithms in prediction of hate speech targets.

Our approach leverages machine learning algorithms such as Decision Tree (DT), Logistic Regression (LR), Naïve Bayes (NB), Random Forest (RF), and Support Vector Machine (SVM). Among these, Naïve Bayes demonstrated the best performance, achieving an accuracy of 93% when using the optimal combination of features. Additionally, we have employed advanced Large Language Models (LLMs) such as DeBERTa v3-large, Mistral 7B, and RoBERTa-large. These LLMs are transformer-based models trained on extensive datasets, allowing them to capture intricate language patterns and nuances. By integrating these LLMs using an ensemble learning approach, we aim to leverage their individual strengths to enhance the prediction accuracy further. The ensemble of LLMs provides a reliable mechanism to handle the varied expressions of hate speech.

Rest of the paper is organized as follows: Section 2 highlights limitations of existing research in detecting hate speech. Section 3 presents the methodology for developing the HSTD dataset. Section 4 presents the proposed HTPK framework for prediction. Section 5 details the experimental setup and comparison with existing methods. Section 6 concludes this research and outlines the directions for future research.

By advancing our understanding of hate speech dynamics and developing reliable predictive models, this research contributes to creating safer online environments and informs social media policies aimed at mitigating hate speech.

## 2 Related work

Research on moderating hateful language on social media platforms has increasingly highlighted the importance of automated hate speech detection, sarcasm, sentiment analysis, and rumors. This body of work utilizes a range of feature extraction and machine learning methods for addressing different facets of language analysis [4], [5], [6], [7], [8], [9], [10].

### 2.1 Feature extraction methods:

• **Token Frequency Features:** Initially, studies employed token frequency features like bag-of-words (BoWs) and dictionaries. However, these methods often failed to capture the contextual nuances of language [11], [12].
• **N-grams:** To address the limitations of BoWs, researchers have utilized n-grams, which have shown improved performance by capturing sequences of words and thus better context [13], [14].
• **TF-IDF** has been widely used to quantify the importance of words within documents of a corpus. It is a widely used measure in text classification [15].
• **Topic Modeling:** Techniques like topic extraction and similarity analysis have been applied to identify and compare themes within text, offering insights into underlying discursive patterns [16].
• **Sentiment-based Features:** These features assess the sentiment of texts, categorizing content into positive, negative, and neutral sentiments, which is crucial for understanding emotional undercurrents in social media posts [5].
• **Linguistic Features:** Advanced linguistic features such as part of speech tagging, named entity recognition, and typed dependencies have been used to refine the understanding of syntactic and semantic structures in text [8], [17].

Despite the theoretical advantages of sophisticated models like BERT, RoBERTa, and DeBERTa [18], [19], [20], studies have shown that simpler, well-tuned models such as Naïve Bayes can sometimes yield superior results, especially in constrained computational settings [7]. This underscores the need for appropriate selection of features and models.

Moreover, the field is also exploring transformer-based architectures and large language models (LLMs) to detect and analyze hate speech across multiple mediums including text, images, memes, audio, and videos [21], [22], [23], [24], [25], [26], [27], [28].

Ambiguity in hate speech definitions continues to challenge the development and effectiveness of detection algorithms, necessitating a varied and adaptive approach as outlined by different platforms and legislative bodies summarized in Table 1.

**Table 1:** Definitions of hate speech.

Source	Definition
Twitter	“Hateful conduct: You may not promote violence against or directly attack or threaten other people on the basis of race, ethnicity, national origin, sexual orientation, gender, gender identity, religious affiliation, age, disability, or serious disease. We also do not allow accounts whose primary purpose is inciting harm towards others on the basis of these categories” a
Facebook	“Direct attack on people-based on what we call protected characteristics – race, ethnicity, national origin, religious affiliation, sexual orientation, caste, sex, gender, gender identity and serious disease or disability. We also provide some protections for immigration status. We define “attack” as violent or dehumanizing speech, statements of inferiority, or calls for exclusion or segregation” b
European Council	“All forms of expression which spread, incite, promote or justify racial hatred, xenophobia, anti-Semitism or other forms of hatred-based on intolerance, intolerance expressed by aggressive nationalism and ethnocentrism, discrimination and hostility towards minorities, migrants and people of immigrant origin” c
Chetty and Alathur	“Hate speech is any speech, which attacks an individual or a group with an intention to hurt or disrespect based on identity of a person” [3]
Nobata et al.	“Language which attacks or demeans a group based on race, ethnic origin, religion, disability, gender, age, disability, or sexual orientation/gender identity” [14]

a https://web.archive.org/web/20240509170900/https://help.twitter.com/en/rules-and-policies/hateful-conduct-policy/, last accessed on Tuesday 5^th^ November, 2024.

b https://web.archive.org/web/20240530101945/https://transparency.meta.com/en-gb/policies/community-standards/hate-speech/, last accessed on Tuesday 5^th^ November, 2024.

c https://web.archive.org/web/20240422215103/https://www.coe.int/en/web/combating-hate-speech/what-is-hate-speech-and-why-is-it-a-problem-, last accessed on Tuesday 5^th^ November, 2024.

### 2.2 Hate speech target identification

However, fewer studies have been conducted for hate speech target identification in social media. [29] conducted a study to detect hate speech targets classes but also captured targets frequency. For this purpose, they use two social media platforms Whisper and Twitter. [30] conducted a study to distinguish between hate speech targets and hate haters. They worked on personality analysis including profile self-presentation, online visibility and activities on Twitter. As a result, they found that hate victims have often verified and older accounts unlike haters accounts which are often newer and unverified. [31] not only identified offensive language but also identified the targets of offensive language. Towards the end, they categorized the targets into three classes: group, individual and other. All these studies are very important in terms of hate speech target identification and hate speech detection. But they have not worked on the full picture of the issue in terms of target prediction. Because these studies identified targets from hater tweets. We have analyzed in our that there are two types of tweets regarding hate speech, which are:

• **Hater Tweets:** These are the tweets in which the hater is expressing his hatred for the target.
• **Target Tweets:** These tweets are posted by the target and the hater is posting his tweets in response to these tweets.

To the best of our knowledge, the **target tweets** have not been considered in these studies [30], [31], in which the hater uses hate speech in response to a victim's tweet. Therefore, it is important to work on these tweets of victims to predict the targets and to determine if the user will become a target of hate speech [32].

### 2.3 Categories of problems related to hate speech

In this literature review, we have studied issues related to hate speech. We categorized them into three problems which are as follows:

*PB1 – Detecting hateful Tweets*: If one looks at hate speech detection issues on Twitter, different studies have proposed approaches to detect or identify hate speech from **hater tweets** in order to address this problem [38]. For this purpose, labeled data is used that contains one, two, three or four classes (e.g. hate, non–hate, neutral).

*PB2 – Detecting and Characterizing Hateful Users*: In this problem hateful users have been categorized and detected on Twitter. For this purpose, the content shared by users, their activities and network structure are examined and these are compared with common users. Consequently, the results from these show that these users have recent accounts, more followees each day and their posts contain more profane and negative words [44], [43].

*PB3 – Identify and Analyze Targets of Hate from Hater Tweet*: This problem is about identifying and analyzing hate speech targets from hater's post(s). The studies addressed this problem by analyzing the hater's posts. [30]. For example, this is a hater's tweet “Nigger is idiot” in this tweet “Niggar” is targeted by the hater.

### 2.4 Problem statement

This study addresses the problem of predicting the targets of hate speech based on their own tweets. This problem (**PB4**) is different from the previous three (BP1, BP2, BP3) and it has not been addressed yet. It was a motivation for us to work on the target's prediction. Because we have not reviewed any work in the literature addressing this particular issue. Table 2 summarizes the problems that have been addressed in existing studies regarding hate speech on social media. To illustrate the concept of these issues, we have described them in Table 3 with examples.

**Table 2:** Comparison of research related to hate speech detection and prediction.

References	PB1: Detecting Hateful Tweets	PB2: Detecting Hateful Users	PB3: Detect or Predict Targets from Haters' Tweets	PB4: Predicting Targets based on own Tweets
[7], [8], [33], [15], [13], [34], [35], [36], [37], [9], [38], [39], [40], [41], [42]	✓			
[43], [44]		✓		
[31], [37], [30]			✓	
Proposed Framework				✓

**Table 3:** Examples of the types of hate speech found in the literature summarized in Table 2 and categorized in Sections 2.3 and 2.4.

Research Problem	Example	Details
PB1: Detecting hateful tweets	user1: “Shut up idiot”	Detect that the tweet by user1 is hateful
PB2: Detecting hateful user	user1's profile	Hateful user is identified based on the profile and tweet of user1
PB3: Detecting targets of hate	user1: “user2 UN-TAG you retard “	Detect that user2 has become a target of hate by user1
PB4: Predicting targets based on own tweets	user3 “I need to learn how to spell Wednesday”	Predict whether user3 will become the target of hate or not

## 3 Dataset

There are already some excellent works on hate speech detection, for which different datasets have been used [35], [45], [46]. Our analysis of them revealed them to be insufficient for predicting hate speech targets. Moreover, existing datasets are geared towards the haters' posts. This meant that we had to develop our own dataset (the HSTD) for target prediction. The statistics of HSTD are shown in Table 4. We have described the dataset collection and annotation in detail in the Section 3.1.

**Table 4:** Statistics of the Hate Speech Target Dataset (HSTD) used in this study.

Users		Tweets	
Targets	1500	Results in hate	1500
Non-Targets	1500	Did not result in hate	1500

### 3.1 Dataset collection and annotation

The collection of hate speech target data is particularly challenging due to the focus of existing datasets on the hate speech initiators. Our initial analysis of related datasets revealed that they were insufficient for our needs, primarily emphasizing the posts of haters rather than those of the targets. We started with the dataset from the study by [30], which provided IDs of hate speech perpetrators. This dataset included 27,330 tweets from 25,278 instigator accounts targeting 22,287 victim accounts. Using this as a base, we tracked these instigator IDs to collect source tweets that received hateful replies.

Using the Twitter API, we collected 3000 tweets, focusing on direct replies where hate speech was directed at another user. The data collection process involved:

1. **Identifying Target Tweets:** We tracked hater IDs and collected tweets they replied to with hate speech.
2. **Ensuring Balance:** For each target tweet, we collected a corresponding non-target tweet from the same user within the same time period.
3. **Exclusion of Verified Users:** We excluded tweets from verified users (with blue ticks) to avoid bias, as they are often targeted based on their public persona.

### 3.2 Annotation process

Tweets labeled as **Targets** were automatically identified by tracking hater IDs and collecting the source tweets. For comparison, we also collected **Non-Target** tweets from the same users' profiles during the same time period in which they became victims of hate speech. Specifically, if we collected two target tweets from a user's profile using the tracking procedure, we also collected two non-target tweets from the same profile within the same timeframe. This approach ensures that the data remains balanced and accounts for any temporal context.

We assumed that tweets without a reply were non-targets, indicating the user was not targeted in those instances. Consequently, we labeled tweets where users became targets of hate speech as “Targets” and tweets where they did not as “Non-Targets.” Ensuring the accuracy and consistency of the dataset was crucial. Our data collection approach inherently ensured accurate labeling of target tweets by directly tracking hater posts.

An unbalanced dataset may negatively impact prediction performance [47]. Hence, we developed HSTD as a balanced dataset, containing 1500 tweets labeled as ‘Target’ and 1500 labeled as ‘Non-Targets’. We ensured that our dataset captured the nuances of language, context, and sarcasm, which are critical for accurate model predictions.

The exclusion of verified users was based on their unique interaction patterns on social media. Verified users often receive hate speech due to their public persona, which may not reflect typical user interactions. By focusing on common users, we aimed to provide a more representative sample of hate speech targeting on the platform.

The HSTD is a balanced, accurate, and representative dataset for studying hate speech targets. By focusing on common users and ensuring comprehensive data collection and annotation processes, we provide a reliable foundation for our hate speech target prediction framework.

**Figure 1:**
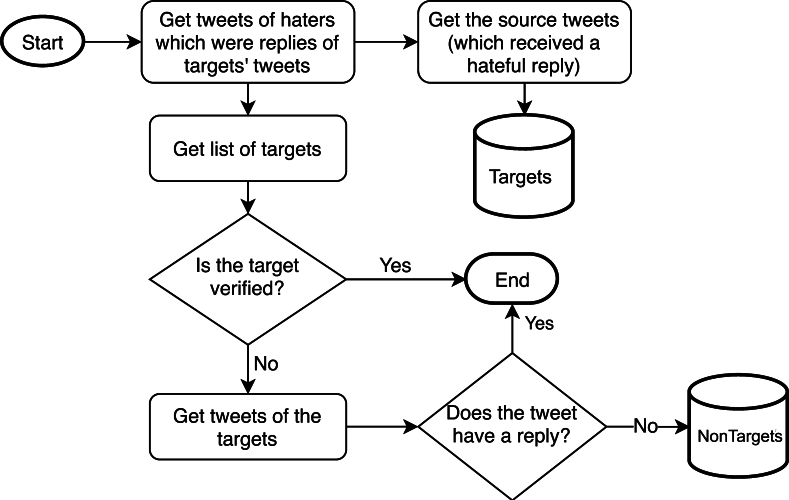
Data Collection Process for Hate Speech Target Dataset (HSTD): This flowchart illustrates the methodology used to collect tweets for the HSTD, including the identification and categorization of target and non-target tweets by tracking hater replies and user profiles.

Fig. 1 illustrates the data collection process for the HSTD.

## 4 Proposed framework (HTPK)

**Figure 2:**
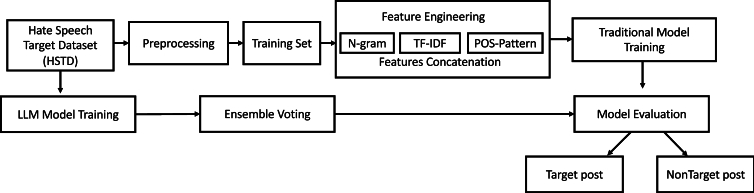
**Hate Speech Target Prediction Framework (HTPK)** Overview of the Hate-speech Target Prediction Framework (HTPK): This diagram illustrates the main components and flow of the HTPK framework. Raw data is collected and preprocessed to form the Hate Speech Target Dataset (HSTD). Traditional machine learning models are trained on features extracted from the preprocessed data, including TF-IDF, N-grams, and PoS tags. Concurrently, large language models (LLMs) are trained directly on the raw text. Predictions from multiple LLMs are combined using an LLM Ensemble Learning approach, with the final evaluation assessing the performance using various metrics.

Predicting the target of hate speech on social media is a challenging task due to the varied ways individuals express themselves, including hate speech. Handwritten rules for hate speech target prediction are impractical due to this diversity. Therefore, we propose the Hate-speech Target Prediction Framework (HTPK) to predict the targets of hate speech on Twitter. The framework consists of five phases, as shown in Fig. 2: raw data collection, preprocessing, feature extraction, model training, and evaluation. Raw data is collected and preprocessed to form the Hate Speech Target Dataset (HSTD). Traditional machine learning models are trained on features extracted from the preprocessed data, while large language models (LLMs) are trained directly on raw text. The predictions from multiple LLMs are combined using an LLM Ensemble Learning approach, followed by a comprehensive evaluation using various performance metrics.

### 4.1 Data pre-processing

Twitter data exhibits substantial language diversity and noise due to its informality, including unknown or useless characters, short comments, and emoticons [48], [49], [50]. To reduce this noise, we apply the following preprocessing steps:

• Convert all text to lowercase.
• Apply stemming to reduce words to their base forms.
• Tokenize the text to split it into individual words.
• Remove irrelevant characters, stop-words, emoticons, punctuation, URLs, numbers, double spaces, and emails.

These steps ensure that the data is clean and suitable for further processing.

### 4.2 Feature extraction

Feature extraction is crucial for improving classifier performance. We extract three types of tweet features and combine them for input into machine learning algorithms, creating a meaningful representation of the tweets.

#### 4.2.1 TFIDF and N-grams based features

The state-of-the-art methods [15] use TF-IDF for representing tweet contents, normalized by L1 or L2 normalization. Furthermore, machine learning algorithms are trained to process natural language [51]. Humans can easily understand natural language, but machine learning algorithms require numeric inputs. Therefore, creating a language pattern that these algorithms can understand is essential. Each word has its specific meaning, but merging words provide better context and understanding. The Bag of Words (BoW) model relies on N-grams (sequences of words derived from text). Word N-grams are based on whole words, while character N-grams are based on characters. By using N-grams, we can anticipate word $t_{n}$ based on preceding n−1 words as shown in Equation (1). We considered unigrams, bigrams, and trigrams in this study.

(1) $$p(t_{n}|t_{1},t_{2},t_{3},\cdots ,t_{i-1})$$

#### 4.2.2 Part of speech tags for pattern extraction

Part of Speech (PoS) tagging is used for analyzing relationships among words and text content. Using PoS, we replace the original words in the tweets with their grammatical positions in the sentence. In PoS tagging, a parse tree is constructed to extract relational patterns among words based on their definitions and context. PoS tagging marks words in tweets with corresponding grammatical tags. More formally, a word *w* is associated with a tag from the set $t_{a},\cdots ,t_{n}$ in the dataset. A tag $t_{g}$ is assigned to a word *w* based on Equation (2).

(2) $$A(t_{g}|w)=\frac{d(w,t_{g})}{d(w,t_{a})+\cdots +d(w,t_{n})}$$

Where $d(w,t_{g})$ is the frequency of the word *w* appearing with the tag $t_{g}$ in the dataset. Thus, $A(t_{g}|w)$ represents the ambiguity of the word. For instance, the word “insult” is a noun and a verb as well, as shown in the following sentences:

• His replies are an **insult** to the targets of war. **(noun)**
• Don't **insult** anyone. **(verb)**

To understand the use of these terms, one must consider both the context and definition of the word. We used Penn Treebank^1^ PoS tags. The words in the dataset are assigned their corresponding tags. A column named PoS tag vectorization is created in the dataset. We extracted the pattern of each tweet from target and non-target classes using PoS tag vectorization. For instance, the following tweet “Name you are an idiot, who told you they wanted to be friends with you” [“NN-Name PRP-you VBP-are JJ-idiot, WP-who VBD-told PRP-you PRP-they VBD-wanted TO-to VB-be NNS-friends IN-with PRP-you”]. This captures syntactical content, and the frequency of the tags in the tweet is used as a feature.

### 4.3 Model training

We used various algorithms for training our models. Each algorithm was selected based on its strengths in text classification tasks. Validation was performed using 10-fold cross-validation to ensure strength and generalizability. Hyperparameter tuning was used for optimizing the performance of each model.

To diversify our model training, we included advanced transformer-based models such as DeBERTa v3-large, Mistral 7B, and RoBERTa-large. Additionally, we implemented a majority vote ensemble learning approach to combine these models, aiming to capitalize on their individual strengths and improve overall prediction accuracy.

### 4.4 Evaluation

We use the measures accuracy, precision, recall, and F1-score for evaluating model performance. Accuracy is the main evaluation measure used for model selection due to its comprehensive reflection of overall performance. This detailed approach ensures that each phase of the HTPK framework is methodically executed, leading to reliable and accurate prediction of hate speech targets on Twitter.

## 5 Experiments

### 5.1 State-of-the-Art

We benchmarked our methods against established studies, specifically the works cited as [15], [13], and [52]. These studies informed our initial approach, especially in the domain of feature selection and model evaluation strategies. Unlike the state-of-the-art methods, which primarily used datasets related to hate speech detection, we applied similar techniques to our own Hate Speech Target Dataset (HSTD), allowing for direct comparisons under comparable experimental conditions. This application on a novel dataset underscores the strength and adaptability of these methods to different yet related tasks.

Furthermore, the study by [15], [52] reports accuracy results, and the [13] study discusses the weighted average of F-measure. The comparison of the models is given in Table 5, where techniques from the literature were applied to HSTD. Consistent preprocessing techniques and feature usage were maintained across all comparisons to ensure methodological integrity. Notably, we emphasize using Part of Speech (PoS) tags in our feature set, which significantly contributed to the model performance by capturing syntactical relationships in the text.

**Table 5:** Performance Comparison of State-of-the-Art Models on HSTD Dataset: This table presents the accuracy scores of various state-of-the-art models applied to the Hate Speech Target Dataset (HSTD). Significant improvements are highlighted, particularly with the Naïve Bayes classifier using word bigram, TFIDF, and PoS tags. The highest scores for each feature combination are bolded, demonstrating superior performance.

Features/Model	SVM	Logistic Regression	Naive Bayes	Random Forest
**Alfina et al. (2017)****[13]**				
Word unigram	86.42	-	**88.78**	82.50
Word bigram	72.42	-	73.14	68.78
Char trigram	58.72	-	58.72	61.45
**Gaydhani et al. (2018)****[15]**				
unigram + L1	89.10	**90.42**	88.44	-
(unigram and bigram) + L1	**90.75**	89.10	89.43	-
(unigram and trigram) + L1	88.11	87.45	89.10	-
(unigram) + L2	89.10	88.77	87.45	-
(unigram and bigram) + L2	89.76	89.76	89.10	-
(unigram and trigram) + L2	88.44	89.10	88.44	-
**F. Haider et al. (2023)****[52]**				
unigram and bigram + TFIDF	89.76	89.76	–	80.85

**Proposed Approach**				
word bigram + TFIDF + PoS Tags	92.40	89.80	**93.10**	87.10

Our results highlight differences from these benchmarks since we employed our own dataset (HSTD). The findings reveal that Na“ive Bayes performs better on the unigram feature. We considered the impact of tweet length, structure, and informality, crucial factors that affect performance when N-gram size increases. Consequently, we observed that increasing N-gram size reduces performance due to these factors. All classifiers perform well with L1 regularization of TF-IDF. However, LR (logistic regression) performs better on unigram and bigram (1,2) with TFIDF L1 than NB and SVM, aligning with recent advancements in feature handling for social media text.

PoS tags enhanced the model performance significantly, as they capture the syntactical context and relationships between words, providing a better understanding of the text. Features used in the state-of-the-art and proposed framework are summarized in Table 6.

**Table 6:** Features used in state-of-the-art methods and the proposed framework.

Reference	N-gram	TF-IDF	PoS Patterns
[13]	✓		
[15]	✓	✓	
[52]	✓	✓	
Proposed Approach	✓	✓	✓

### 5.2 Additional models and ensemble learning:

We extended our experiments by incorporating advanced transformer models including DeBERTa v3-large, Mistral 7B, and RoBERTa-large. These models were fine-tuned on our Hate Speech Target Dataset (HSTD) to predict potential targets of hate speech. Additionally, we employed majority voting ensemble learning techniques to combine the predictions from these models, aiming to improve overall accuracy

### 5.3 Results and discussion

After feature extraction, where we concatenated features (TFIDF, N-grams, PoS tags), giving each feature equal priority to ensure a balanced representation of the tweet data, after parameter tuning, we moved to our final experiments, performed using Scikit-learn. Scikit-learn offers a variety of classifiers categorized by algorithm type (e.g., rule-based, decision tree-based, etc.). We considered five well-known machine learning algorithms for prediction: Logistic Regression, Support Vector Machines, Naïve Bayes, Random Forest, and Decision Trees. Each model was trained using 10-fold validation on the HSTD, and their performance was assessed using key measures including accuracy, recall, precision, and F1 scores.

We have used a combination of different features on HSTD. These features have been combined using Equation (3).

(3) $$F_{u}=\{f_{1},f_{2},f_{3}\}$$

Where $f_{u}$ denotes features union. $f_{1}$, $f_{2}$, and $f_{3}$ are defined in Equation (4), Equation (5), and Equation (6), respectively. Through ((4) we have predicted the probability of words in the dataset.

(4) $$f_{1}=p(t_{n}|t_{1},t_{2},t_{3},\cdots ,t_{i-1})$$

Formally, $f_{2}$ is a second feature. tfidf indicates termFrequency-InverseDocuments frequency. Additionally, the *t* term in dataset *D* can be calculated as follows (5):

(5) $$f_{2}=tfidf(t,d)=tf(d,t)⁎idf(d,t)$$

formally, *w* denotes the words in dataset with $t_{a},....t_{n}$ tags. Tags have been assigned by using Equation (6). Where $d(w,t_{g})$ is the frequency of $t_{g}$ and *w* appears in the tweets dataset (HSDT). Furthermore, $A(t_{g}|w)$ is probably context.

(6) $$f_{3}=A(t_{g}|w)=\frac{d(w,t_{g})}{d(w,t_{a})+\cdots +d(w,t_{n})}$$

Table 7, presents the accuracy results for various combinations of features across different machine learning classifiers. We observed that word unigram with TFIDF and PoS tags shows the best results for lower values of N, probably because of the tweet's short length, lack of structure, and informality, along with the presence of diminutives and typos. Conversely, increasing the range of character trigrams (n=1–3) reduces performance, possibly due to the increased complexity and overfitting. Results in bold refer to the best result of experiments. Target prediction improved by the features combination, with improvements of up to 3% and 5% as compared to state-of-the-art methods [13], [15], [52].

**Table 7:** Accuracy results for various combinations of features across different machine learning classifiers. The table highlights the superior performance of the Naïve Bayes classifier.

Features	SVM	DT	RF	LR	NB
word unigram + TFIDF + PoS Tags	.848	.855	.871	.898	.911
word bigram + TFIDF + PoS Tags	.898	.848	.861	.924	**.931**
char trigram + TFIDF + PoS Tags	.894	.818	.865	.888	.904

Table 9 further details the recall, precision, and F-measure for all classifiers with the best feature combination (TFIDF, PoS tags, and bigram). Notably, the Naïve Bayes classifier consistently outperformed others in terms of accuracy, highlighting its strengths in text classification tasks.

The results indicate that feature selection plays a crucial role in model performance. The combination of PoS tags with TFIDF and N-grams provided the best results, demonstrating the importance of capturing syntactical relationships in text data. Our findings also show that increasing the dataset size improves model performance across all classifiers, underscoring the value of large, high-quality datasets in machine learning.

In addition, tuning the parameters of NB also improves accuracy. When viewed in terms of precision, NB performance is lower than LR. It is examined that the recall for non-target tweets is comparatively low at 0.92. This means that 8% of actually non-target tweets were incorrectly classified by the NB. In addition, the target class precision is 0.92 which means that there were 8% tweets that were actually non-target and were classified as target. On the contrary, the precision for the non-target class and the recall for the target class is 0.93, which is considerably better.

**Figure 3:**
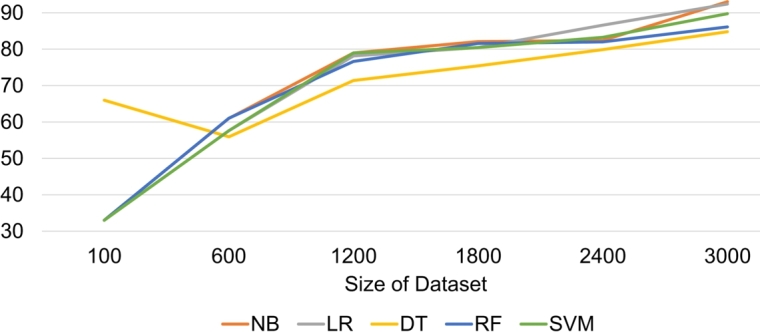
Accuracy of HTPK Framework Across Different Dataset Sizes. This graph illustrates the impact of dataset size on the accuracy of various classifiers within the HTPK framework.

If we view LR performance, its performance in terms of accuracy is less than NB but its performance is better than other classifiers. After completing experiments, we analyzed that Decision Tree performs poorly as compared to Logistic Regression, Naïve Bayes, Random Forest Tree, and SVM. This is because all the features of a large tree size need to be included. Due to the size of the tree, the classifier requires crossing multiple nodes till it arrives at the leaf node and anticipates the target and non-target classes. This increases the likelihood of errors due to the long path consequently reducing classifier accuracy. As Fig. 3 shows, as the size of the dataset increases, the performance of DT and RF decreases. We have used different sizes of the dataset on HTPK as can be seen in Fig. 3. The purpose of using different sizes is to determine the effectiveness of various data sizes for ML algorithms. We have found that increasing the dataset size improves the performance of HTPK.

### 5.4 Performance of advanced models and LLM fusion ensemble

Table 8 presents the ensemble model combining DeBERTa v3-large, Mistral 7B, and RoBERTa-large with an accuracy of 89.29%. While this is an impressive result, it did not surpass the Naïve Bayes classifier's performance of 93%. This suggests that while transformer-based models are powerful, their performance may be limited by the dataset size and the specific characteristics of the task. The marginal improvement from the ensemble model highlights the need for further fine-tuning and possibly larger datasets for best utilizing these advanced models.

**Table 8:** Performance of Various Models and Ensemble Learning: This table presents the accuracy of DeBERTa v3-large, Mistral 7B, and RoBERTa-large models individually and in combination using an ensemble learning approach. The ensemble method achieved an accuracy of 89.29%, slightly lower than the Naïve Bayes classifier's performance but demonstrating the strength of transformer-based models.

Model	Accuracy (%)
RoBERTa-large	89.12
DeBERTa v3-large	88.63
Mistral 7B	85.99
+ Ensemble	89.29

**Table 9:** Precision (P), recall (R), F1-Measure (F1), and accuracy (Acc) for different classifiers predicting hate speech targets.

Classifiers	Target Class			Non-Target Class			Accuracy (%)
	P	R	F1	P	R	F1	
**NB**	0.92	0.93	0.93	0.93	0.92	0.92	**.93**
**DT**	0.83	0.83	0.84	0.83	0.83	0.83	.85
**RF**	0.94	0.79	0.86	0.81	0.95	0.87	.86
**SVM**	0.91	0.87	0.89	0.87	0.91	0.89	.90
**LR**	0.95	0.89	0.92	0.89	0.95	0.92	.92

In summary, the HTPK allows us to effectively predict targets of hate speech. As far as we know, it is the first framework to do this based on common user tweets. We expect that the predictions can be made useful by suggesting whether a post is likely to elicit hate speech to a potential target user.

### 5.5 Characterizing target and non-target posts

This research investigates why certain individuals become targets of hate speech. To this end, we analyzed the tweet contexts of common users, observing that word choice is crucial in determining whether a post might provoke hate speech. Our analysis revealed that posts containing negative words,^2^ promoting hate speech, attacking personalities, criticizing celebrities, defending racism, expressing anger, or misrepresenting truth are more likely to result in the user becoming a victim of hate speech.

**Figure 4:**
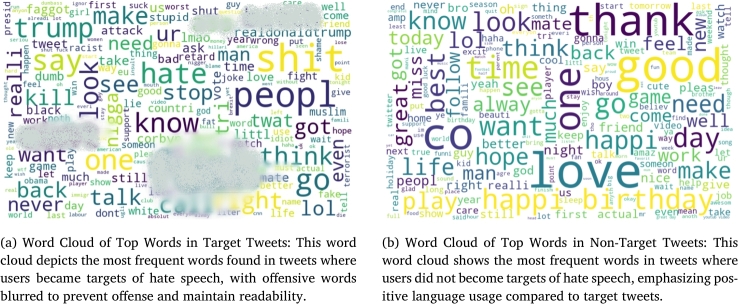
Overall Comparison of Language in Target and Non-Target Tweets: This comparison highlights the differences in language used in target versus non-target tweets, demonstrating the importance of word choice in becoming a victim of hate speech.

In Fig. 4a, we present a word cloud showing the most common words in posts of users who became targets of hate speech. Offensive words have been blurred to avoid offending readers and maintain readability. This visualization clearly indicates the prevalence of abusive and negative language in target tweets.

Conversely, Fig. 4b depicts the word cloud for non-target tweets, highlighting more positive and respectful language. These tweets show respect, promote love, and contain fewer words associated with anger, shame, and insult.

We further analyzed the general words used in both target and non-target tweets and found that they are often similar; however, their context and connotations differ significantly. For instance, the word “scream” in “I want to scream” (non-target) versus “you die soon” (target) illustrates how negative context and direct threats contribute to becoming a target.

We provide specific examples to illustrate these points:

• Non-Target: “I want to scream” (user1)
• **Target:** “you die soon” (user1)

These examples demonstrate that the content of posts significantly influences whether a user becomes a target of hate speech. The words users choose in their posts reflect their personalities and can impact how others react to them on social media. Therefore, careful word selection is crucial in preventing hate speech victimization.

## 6 Conclusion

This study addressed the critical task of predicting hate speech targets on Twitter using machine learning. We developed the novel Hate Speech Target Dataset (HSTD), which is the first dataset specifically focused on the posts that lead to users becoming targets of hate speech. Alongside this, we proposed a novel framework, HTPK, for predicting these targets.

Our experiments evaluated the effectiveness of the HTPK framework using various machine learning algorithms. Naïve Bayes consistently outperformed the other models, particularly when utilizing a combination of Part of Speech (PoS) tags, TFIDF, and N-grams (1 to 2). The experiments also demonstrated that larger dataset sizes generally improve model performance, underscoring the importance of extensive, high-quality datasets in machine learning.

Our experiments with advanced transformer models and ensemble learning show promising results, with the ensemble achieving an accuracy of 89.29%. Although this is slightly lower than our best-performing Naïve Bayes classifier, it underscores the potential of leveraging state-of-the-art models for hate speech target prediction. Future work will explore larger datasets and more sophisticated ensemble methods to further enhance prediction accuracy.

The findings reveal that the language used in posts significantly influences whether a user becomes a target of hate speech. Posts with negative, abusive, or controversial language are more likely to provoke hate speech responses. This highlights the critical role of word choice in social media interactions. Both content creators and social media platforms have roles to play in mitigating hate speech. Platforms should enhance their moderation tools, while users should be aware of the potential consequences of their language choices.

### 6.1 Future work

Future research will explore extending the HTPK framework to include real-time prediction capabilities and incorporate multimodal data such as images and videos to enhance prediction accuracy. Additionally, studies into the psychological impacts of targeted hate speech can help develop more nuanced preventive strategies. Further investigation into adaptive algorithms that evolve with changing social media dynamics will also be crucial.

## CRediT authorship contribution statement

**Sahrish Khan:** Writing – review & editing, Writing – original draft, Visualization, Validation, Software, Resources, Methodology, Investigation, Formal analysis, Data curation, Conceptualization. **Rabeeh Ayaz Abbasi:** Writing – review & editing, Writing – original draft, Validation, Supervision, Project administration, Methodology, Formal analysis, Conceptualization. **Muddassar Azam Sindhu:** Writing – review & editing, Validation, Formal analysis. **Sachi Arafat:** Writing – review & editing, Validation. **Akmal Saeed Khattak:** Writing – review & editing, Writing – original draft, Methodology, Investigation, Formal analysis, Conceptualization. **Ali Daud:** Writing – review & editing, Writing – original draft, Validation. **Mubashar Mushtaq:** Writing – review & editing.

## Declaration of Competing Interest

The authors declare that they have no known competing financial interests or personal relationships that could have appeared to influence the work reported in this paper.

## Data Availability

Data and code will be made available on request.

## Footnotes

1 https://web.archive.org/web/20240423195602/https://www.ling.upenn.edu/courses/Fall_2003/ling001/penn_treebank_pos.html, last accessed on Tuesday 5^th^ November, 2024.

2 https://web.archive.org/web/20240417115711/https://github.com/LDNOOBW/List-of-Dirty-Naughty-Obscene-and-Otherwise-Bad-Words, last accessed on Tuesday 5^th^ November, 2024.
